# Deep Learning Prediction Model for Patient Survival Outcomes in Palliative Care Using Actigraphy Data and Clinical Information

**DOI:** 10.3390/cancers15082232

**Published:** 2023-04-10

**Authors:** Yaoru Huang, Nidita Roy, Eshita Dhar, Umashankar Upadhyay, Muhammad Ashad Kabir, Mohy Uddin, Ching-Li Tseng, Shabbir Syed-Abdul

**Affiliations:** 1 Department of Radiation Oncology, Taipei Medical University Hospital, Taipei 110, Taiwan; 2 Graduate Institute of Biomedical Materials and Tissue Engineering, College of Biomedical Engineering, Taipei Medical University, Taipei 110, Taiwan; 3 Department of Computer Science and Engineering, Chittagong University of Engineering and Technology, Chittagong 4349, Bangladesh; 4 Graduate Institute of Biomedical Informatics, College of Medical Sciences and Technology, Taipei Medical University, Taipei 106, Taiwan; 5 International Center for Health Information Technology, College of Medical Science and Technology, Taipei Medical University, Taipei 106, Taiwan; 6 Faculty of Applied Sciences and Biotechnology, Shoolini University of Biotechnology and Management Sciences, Solan 173229, Himachal Pradesh, India; 7 School of Computing, Mathematics and Engineering, Charles Sturt University, Bathurst, NSW 2678, Australia; 8 Research Quality Management Section, King Abdullah International Medical Research Center, King Saud bin Abdulaziz University for Health Sciences, Ministry of National Guard—Health Affairs, Riyadh 11481, Saudi Arabia; 9 International Ph.D. Program in Biomedical Engineering, College of Biomedical Engineering, Taipei Medical University, Taipei 110, Taiwan; 10 School of Gerontology and Long-Term Care, College of Nursing, Taipei Medical University, Taipei 110, Taiwan

**Keywords:** survival prediction, patient monitoring, wearables, prognosis, deep learning, decision making

## Abstract

**Simple Summary:**

Palliative care is a vital aspect of healthcare that aims to improve the quality of life for individuals battling life-threatening diseases, such as cancer. Our research delved into the potential of deep learning (DL) model approaches to predict survival outcomes for end-stage cancer patients. Furthermore, we compared the results of wearable-technology-based activity monitoring with traditional prognostic tools. Interestingly, we found that models trained using both clinical data and time series data demonstrated better performance than those trained solely with time series data. Our research findings are novel in the palliative care field since DL models are not typically employed for predicting survival outcomes.

**Abstract:**

(1) Background: Predicting the survival of patients in end-of-life care is crucial, and evaluating their performance status is a key factor in determining their likelihood of survival. However, the current traditional methods for predicting survival are limited due to their subjective nature. Wearable technology that provides continuous patient monitoring is a more favorable approach for predicting survival outcomes among palliative care patients. (2) Aims and objectives: In this study, we aimed to explore the potential of using deep learning (DL) model approaches to predict the survival outcomes of end-stage cancer patients. Furthermore, we also aimed to compare the accuracy of our proposed activity monitoring and survival prediction model with traditional prognostic tools, such as the Karnofsky Performance Scale (KPS) and the Palliative Performance Index (PPI). (3) Method: This study recruited 78 patients from the Taipei Medical University Hospital’s palliative care unit, with 66 (39 male and 27 female) patients eventually being included in our DL model for predicting their survival outcomes. (4) Results: The KPS and PPI demonstrated an overall accuracy of 0.833 and 0.615, respectively. In comparison, the actigraphy data exhibited a higher accuracy at 0.893, while the accuracy of the wearable data combined with clinical information was even better, at 0.924. (5) Conclusion: Our study highlights the significance of incorporating clinical data alongside wearable sensors to predict prognosis. Our findings suggest that 48 h of data is sufficient for accurate predictions. The integration of wearable technology and the prediction model in palliative care has the potential to improve decision making for healthcare providers and can provide better support for patients and their families. The outcomes of this study can possibly contribute to the development of personalized and patient-centered end-of-life care plans in clinical practice.

## 1. Introduction

Palliative care is a holistic approach that addresses not only the physical symptoms but also the emotional, social, and spiritual needs of both patients and their families. The overall goal of palliative care is to enhance the quality of life of patients facing serious illnesses by providing them with the required comfort and support. This can be accomplished via the early diagnosis, assessment, and management of pain along with other symptoms, which are tackled through palliative care [1,2]. According to global disease statistics, cardiovascular disease accounts for 38.5%, cancer for 34%, chronic respiratory disease for 10.3%, AIDS for 5.7%, and diabetes for 4.6% of patients who need palliative care [2]. In the United States, cancer is the most common primary diagnosis among patients with health insurance obtaining hospice care, accounting for 29.6%. After that, the 2nd and 3rd most prevalent diagnoses are heart disease (17.4%) and dementia (15.6%) [3]. These statistics emphasize the importance of palliative care in treating patients’ symptoms and enhancing the quality of life of patients suffering from terminal diseases.

In hospice care, accurate survival estimates are critical for making informed treatment decisions. This becomes even more important when patients with advanced terminal cancer reach the ends of their lives, as this time is critical for evaluating treatment goals and focusing on palliative care [4]. The estimation of survival (prognostication) is a key component of cancer patients’ management, especially for those patients who are at an advanced cancer stage, and has implications for decision making and planning for the patients themselves/their families as well as for the healthcare professionals treating them [4]. The prognoses for advanced terminal cancer patients are not sufficiently accurate in clinical settings, as healthcare professionals are not always good at prognosticating, i.e., predicting whether a patient will live for a couple of months or longer, and they often overestimate their survival [5,6]. To address this issue, various prognostication tools, such as Performance Status (PS), are used in clinical settings to improve prognostication in cancer patients [7]. PS is utilized to determine patients’ abilities to perform their daily tasks and is widely used to describe the statuses of patients’ symptoms and functions according to their ambulatory care needs. The PS score is an assessment of a patient’s capacity to carry out certain daily tasks without assistance, known as activities of daily living (ADLs) [7]. These ADLs can range from fundamental tasks, such as dressing, eating, and bathing, to more intricate activities, such as completing household chores or maintaining regular employment [7].

The related literature has shown PS to be a reliable indicator of survival outcomes in cancer patients [8,9]. Numerous studies have demonstrated that PS evaluations along with clinical symptoms and signs can improve survival prediction in cancer patients [4,10,11,12]. PS has also been frequently employed as a criterion for evaluating a patient’s suitability for participation in clinical trials and modifying treatment strategies [8,9]. Several tools, such as the Eastern Cooperative Oncology Group (ECOG), Palliative Performance Scale (PPS), and Karnofsky Performance Status (KPS) have been used in the assessment of prognoses in terminally ill patients [7,9,13]. Different studies have also established a relationship between cancer patient survival and PS [14,15,16]. However, issues with intra- and inter-observer bias in clinicians can impact the accuracy of PS evaluations [17]. Although Clinician Prediction of Survival (CPS) is more intuitive in clinical practice, it is criticized for clinicians’ tendencies to overestimate patients’ survival, often providing overly optimistic estimates to patients [5,6,11,18]. Most of the existing integrated prognostication tools are based on logistic regression analysis that has been successful at predicting the short-term mortality (up to six months) of patients [19,20]. However, the utilization of machine learning (ML) technology could result in improved prognostication by considering a multitude of variables and their relationships, both linear and nonlinear [21,22,23]. The studies by Arkin et al. [24] and Manz et al. [25] both managed to show improvement in the abilities of ML approaches to predict survival among cancer patients when compared with statistical methods.

Healthcare has been transformed by the development of wearable technology. This technology utilizes wearable devices, sensors, mobile applications, and tracking technologies, has enormous applications in the healthcare domain ranging from patient care to personal health, and is absolutely necessary for the diagnosis, prevention, monitoring, and treatment of chronic diseases [26]. This cutting-edge technology has opened up new avenues for healthcare providers to track changes in patients’ activity levels and gain valuable insights into their physical health. One of the important applications of this technology in critical settings such as palliative care is in the real-time monitoring of patients’ activities in three dimensions: acceleration, angle, and spin [27]. Recently, in our previous study, we evaluated the feasibility of using actigraphy-based patient monitoring to predict survival outcomes and found that wearable devices (WDs) can be useful prognostic tools for palliative care patients nearing the end of their lives. These devices reported greater angle and spin movements as early as in the first 48 h of observation in cancer patients who were still alive following discharge from a hospice inpatient unit [28,29]. Applied research has explored the applications of ML techniques for health monitoring, elderly care, and fitness tracking in the last decade and is growing over time [30]. The literature has shown that AI with wearable technology can provide intelligent frameworks with automated solutions to clinicians for the diagnosis, monitoring, and treatment of patients, especially elderly/critical patients [31]. This combination of wearable technology with AI-enabled digital health platforms such as ML algorithms can autonomously measure the changes in the activity and behavior of patients and can serve as a useful tool for proactive interventions in critical care settings such as palliative care [32]. Due to the ability to automatically extract the relevant features required for a given task from high-dimensional and heterogeneous data, the field of deep learning (DL) holds huge potential in the field of predictive, preventive, and precision medicine [33]. The integration of wearable technology and DL survival prediction models in end-of-life care can improve decision making for healthcare providers and provide better support for patients and their families [4,34]. The data collected with wearable devices provide objective information that can be utilized for DL models to predict various patient health conditions and outcomes, such as the in-hospital mortality of end-stage cancer patients during their stays in hospitals [35,36]. For this purpose, we developed a DL-based prediction model to predict survival outcomes. The primary aim of this study was to create this model to analyze both actigraphy data and clinical information in order to predict the survival outcomes of patients. In addition, this study had a secondary aim of comparing the accuracy of the proposed activity monitoring and survival prediction model with traditional prognostic tools, such as the KPS and PPI.

## 2. Materials and Methods

### 2.1. Study Design, Setting, and Recruitment

This study was conducted in the hospice care ward of Taipei Medical University Hospital (TMUH) from 11 December 2019 to 30 June 2022 in Taiwan. This was a prospective observational study conducted in the hospice/palliative care ward of TMUH. The Taipei Medical University Joint Institutional Review Board authorized this trial investigation and approved the study protocol (TMU-JIRB no. N201910041).

The inclusion and exclusion criteria are detailed below.

The inclusion criteria for the recruitment included the following:

(1) Patients above the age of 20;
(2) Confirmed terminal-stage solid cancer diagnosis by two oncologists;
(3) Patients who had given consent for hospice treatment and do-not-resuscitate consent.

The exclusion criteria included the following:

(1) Patients who had signs of dying within a day from admission;
(2) Cancer of unknown origin;
(3) Patients moved to another ward after admission.

If a patient was unconscious and/or unable to articulate themselves effectively, a written agreement for participation needed to be signed by their next of kin. Patients were provided with the flexibility of resigning from this research at any moment, and their data would be deleted in that case.

### 2.2. Data Collection and Acquisition Using Wrist Actigraphy

In this research, an actigraphy device (model no. XB40ACT) from the K and Y Lab at National Yang-Ming University in Taipei, Taiwan was utilized. This particular device, which was validated in a previous study, had dimensions of 4.4 × 1.9 × 0.8 cm and weighed 7 g [37]. It recorded hand motion data in three dimensions every second and translated them into three statistical parameters: activity level, angle, and spin. Due to its battery life of only 14 days, the device transmitted the collected data to a server once a week using Bluetooth technology.

During this study, actigraphy devices were worn on the patients’ wrists using silicon wristbands until they were discharged or passed away. The patients’ activity data were continuously recorded with the devices, 24 h a day, 7 days a week throughout their hospitalization and were wirelessly transmitted via a synchronized mobile application. If a patient’s hospitalization exceeded 10 days, a 2nd wearable device was given on the 11th or 12th day and then collected upon discharge or death.

The clinical data on the patients comprised various parameters, such as the dates of admission and discharge, status at discharge, medications administered during hospitalization, length of stay, gender, age, comorbidities, and diagnosis, all of which were associated with the patients and device identification numbers. If physicians had assessed performance scores for cancer patients’ prognoses upon admission, these values were not incorporated into the data utilized for constructing the models.

### 2.3. Data Processing

The actigraphy device gathered time series data with three components: physical activity, angle, and spin. To handle differences in the lengths of the data collected from each patient, zero padding was used to reach the maximum length of the time series. The class label for expired patients was 0, while for those who may be discharged (MBD), the label was 1.

#### 2.3.1. For 48 h Data

The actigraphy gadget recorded time series data for 66 patients, after eliminating the insufficient data for 2 patients (patient numbers 72 and 61). The time series data lengths varied, with the shortest being 855 for patient number 7 and the longest being 17,607 for patient number 53. To ensure consistency, a fixed length of 9640 was chosen for each patient’s time series data. For patients with data longer than 9640, they were decreased to 9640; for those with shorter data, 0 padding was added. The clinical data for patient numbers 72 and 61 were also excluded as they had too little time series data. The lengths were 58 and 195, respectively.

#### 2.3.2. For 24 h Data

To maintain consistency, a fixed length of 2540 was applied to all patients. For those with longer data, they were reduced to 2540; for those with shorter data, 0s were added to reach a total length of 2540. Additionally, the data for patient numbers 72 and 61 were removed, as performed previously.

#### 2.3.3. For 12 h Data

To maintain the consistency of the time series data for each patient, we established a fixed length of 1120. In cases where the data length was greater than 1120, it was shortened to match the fixed length. In contrast, for patients with data lengths of less than 1120, 0 s were added. Similar to before, the data for patient numbers 72 and 61 were also deleted. Furthermore, early-stage data were used to decrease the time frame from 48 h to 24 and 12 h, and a mean of 20 timesteps was chosen as the average value for each of the 3 time frames (48 h, 24 h, and 12 h).

### 2.4. Data Splitting

The dataset was too small to be partitioned, so a leave-one-out cross-validation (CV) method was applied. This method involved using each instance in the dataset as a test set once, with all other instances used as the training set. This meant that the model was trained and evaluated 65 times, with each evaluation using a single sample for testing and 15% of the remaining data points for validation.

### 2.5. Development of Deep Learning (DL) Model

**Figure 1:**
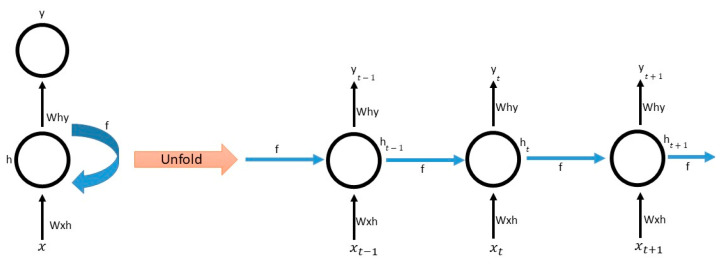
Architecture of RNN models. Note: Each state (*t*) has an input (*x*) and an output (*f*) from a previous state, which are processed in a hidden cell (*h*) to produce the final output (*y*).

**Figure A1:**
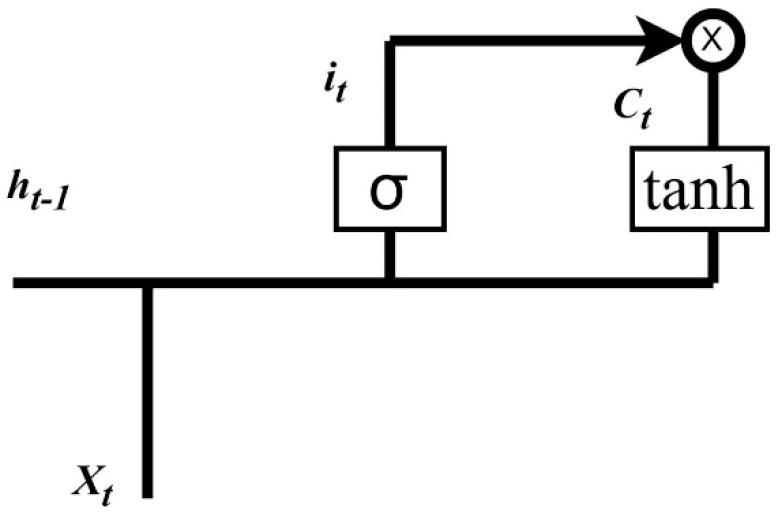
An LSTM unit.

**Figure A2:**
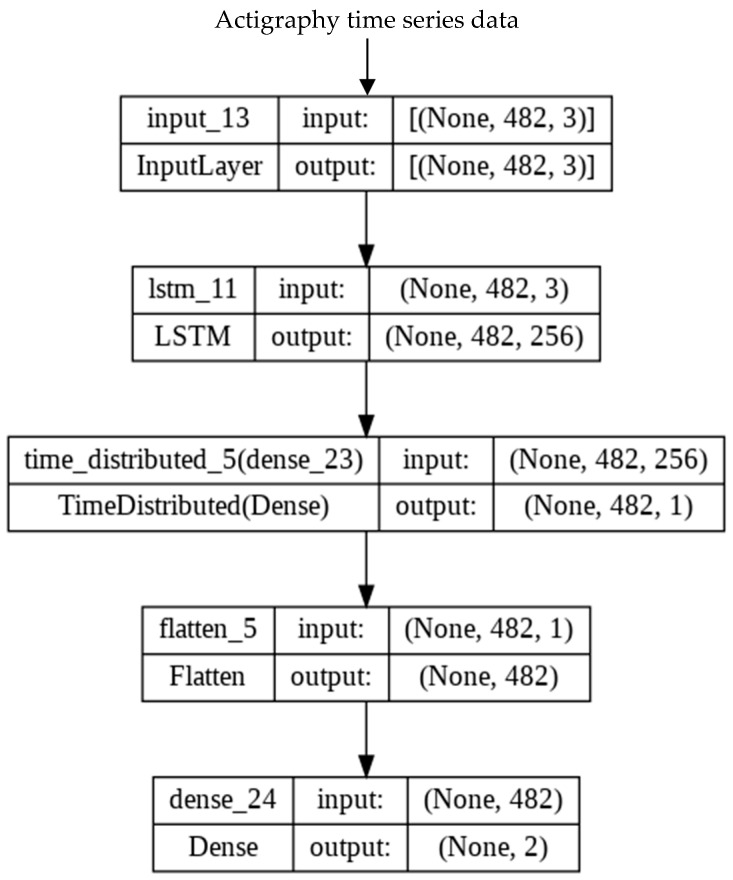
Configuration of LSTM model used for experiment with only time series data.

**Figure A3:**
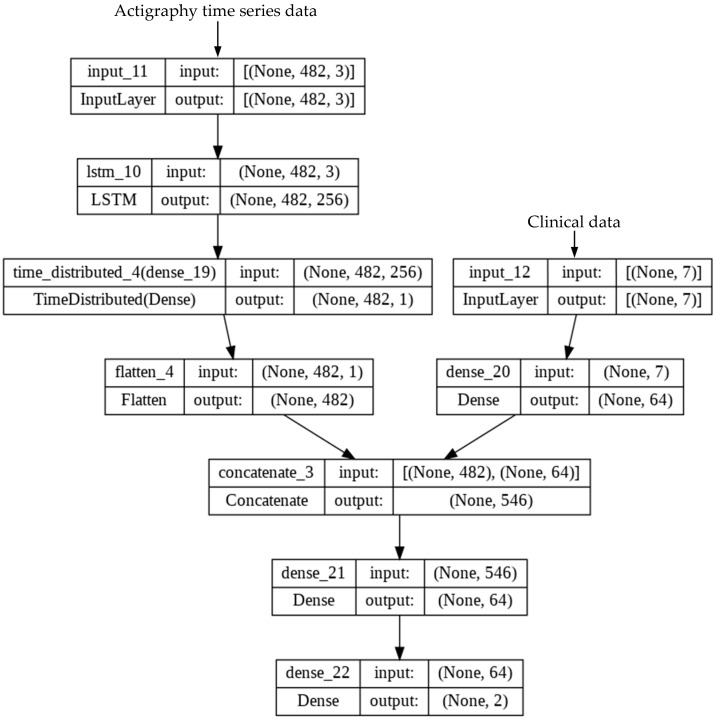
Configuration of LSTM model used for experiment with time series data and clinical data.

**Figure A4:**
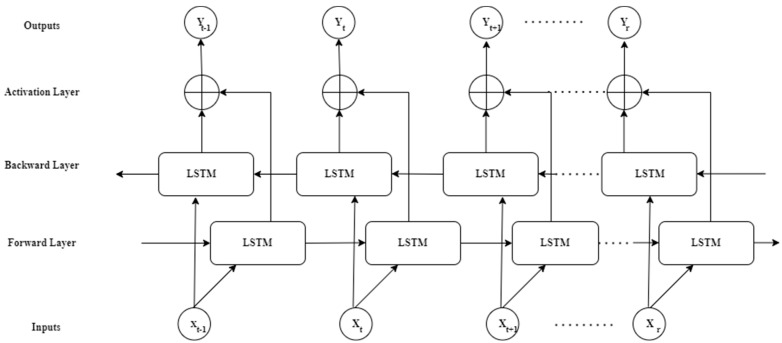
Model architecture of BiLSTM.

**Figure A5:**
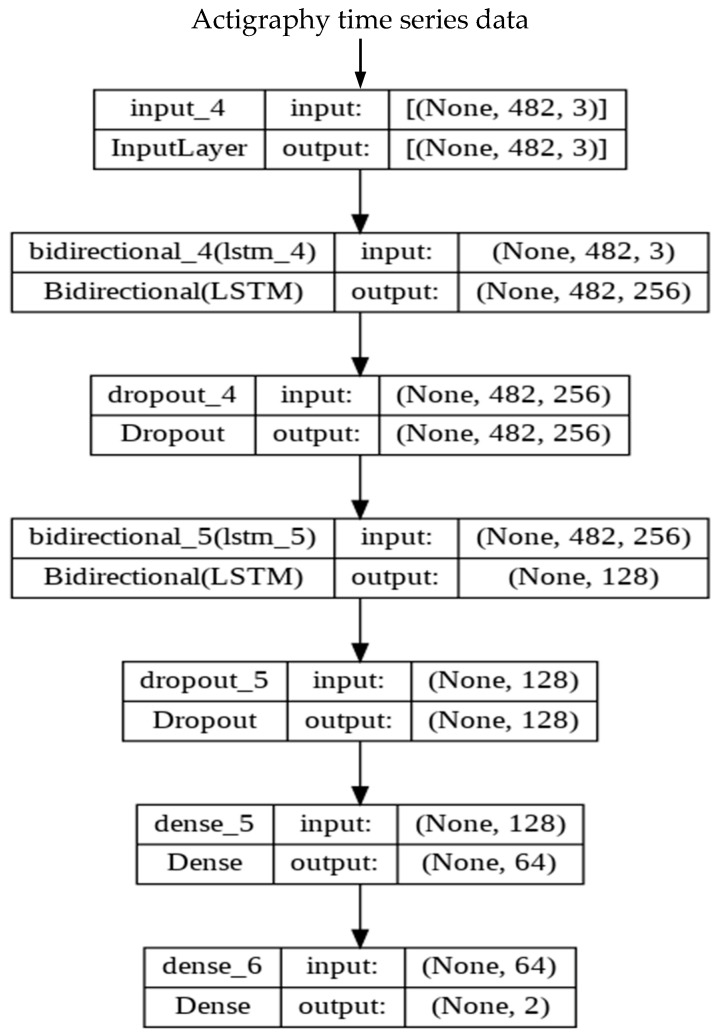
Configuration of BILSTM model used for experiment with only time series data.

**Figure A6:**
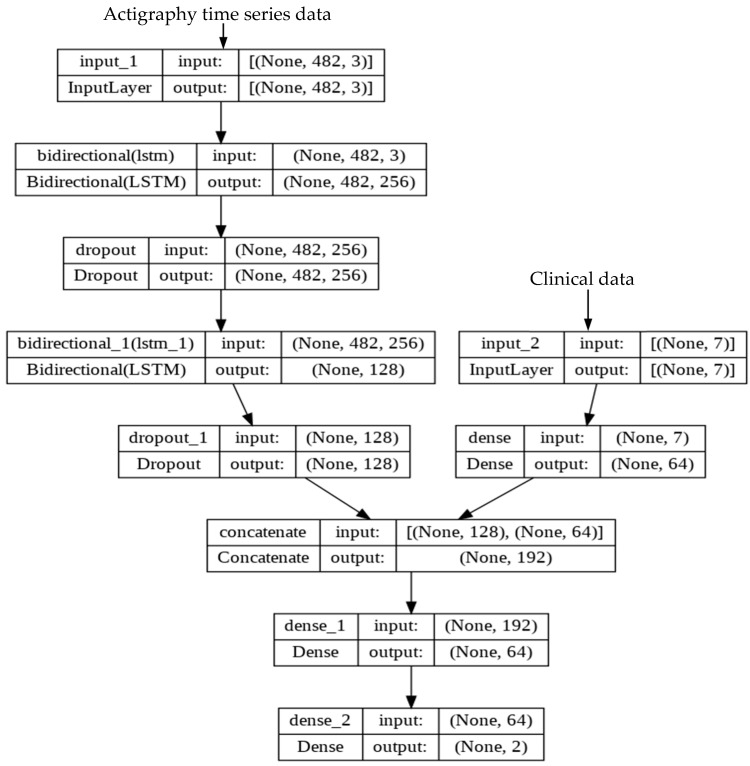
Configuration of BiLSTM model used for experiment with time series data and clinical data.

**Figure A7:**
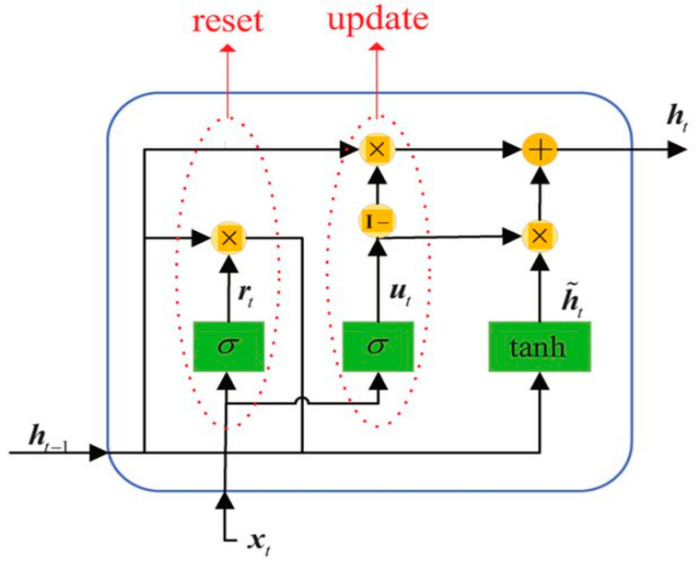
Basic architecture of GRU.

**Figure A8:**
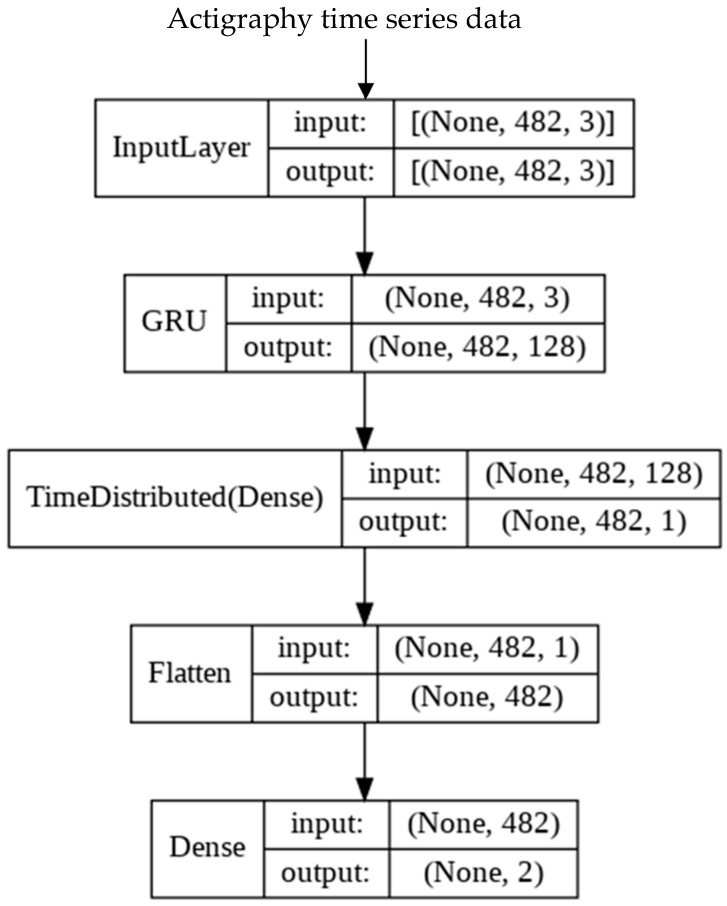
Configuration of GRU Model used for experiment with time series data.

**Figure A9:**
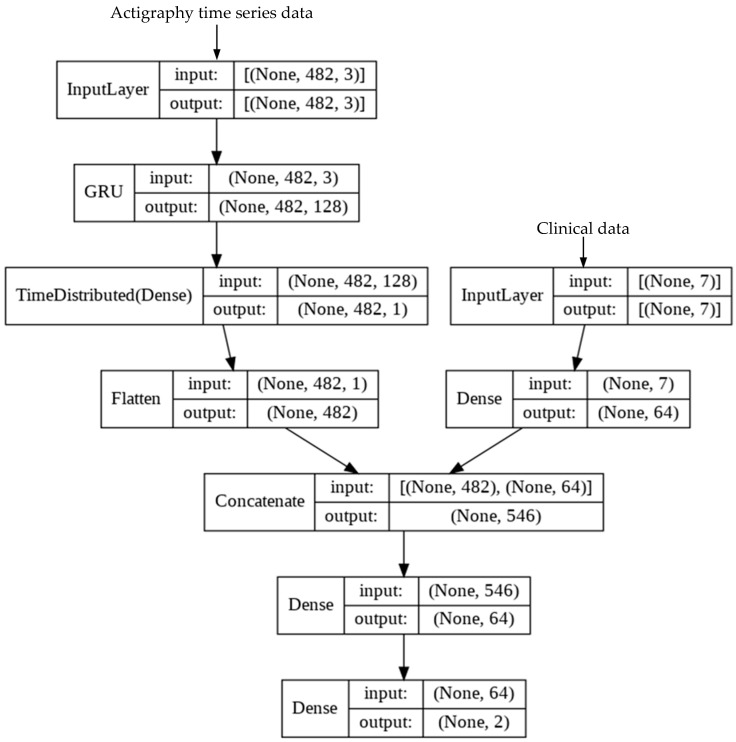
Configuration of GRU model used for experiment with time series data and clinical data.

In this study, four different neural network models were trained to predict the patients’ survival statuses, either MBD or death. The models included a transformer [38], Long Short-Term Memory (LSTM) [39], Bidirectional LSTM (BiLSTM) [40], and Gated Recurrent Units (GRUs) [41]. LSTM, BiLSTM, and GRU are commonly used Recurrent Neural Network (RNN) models that are best utilized for sequential data processing. RNNs are a type of neural network that is specifically designed to handle sequential data, such as time series data, text, or speech (see Figure 1). These models were trained using patients’ clinical data as well as wearable data to determine the best-performing model for accurately predicting the patients’ survival statuses. A brief introduction to the LSTM, BiLSTM, and GRU models is given in the Appendix A (Figure A1, Figure A2, Figure A3, Figure A4, Figure A5, Figure A6, Figure A7, Figure A8 and Figure A9).

A transformer is a DL model architecture for natural language processing (NLP) tasks based on the concept of self-attention, which allows the model to weigh the importance of different parts of an input sequence when making predictions. However, it is different from the previously described sequence-to-sequence models because it does not employ any recurrent networks, e.g., GRU and LSTM. The transformer model [38] is based on the attention mechanism, which means that the weights depend on how a feature of a sequence (represented by the letter Q) interacts with all the other elements in the sequence (represented by K) (see Equation (1)). The weights are also given a distribution between 0 and 1 using the SoftMax function. We used a multi-head attention layer, which consists of different layers running in parallel.

(1) $$Attention\;\left(Q,\;K,\;V\right)=softmax\;\left(\frac{QK^{T}}{\sqrt{d_{k}}}\right)V,$$

**Figure 2:**
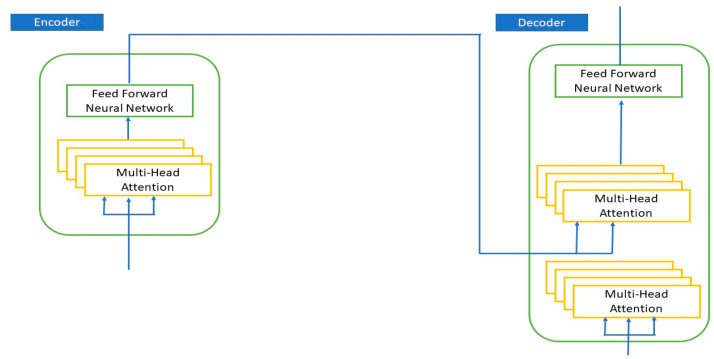
The encoder–decoder structure of the transformer architecture.

A transformer is a structure for converting one sequence into another with the aid of two components, an encoder and a decoder (see Figure 2) [38]. The encoder and decoder blocks are composed of some Multi-Head Attention and Feed-Forward Networks. The best features are chosen via Feed-Forward Networks.

**Figure 3:**
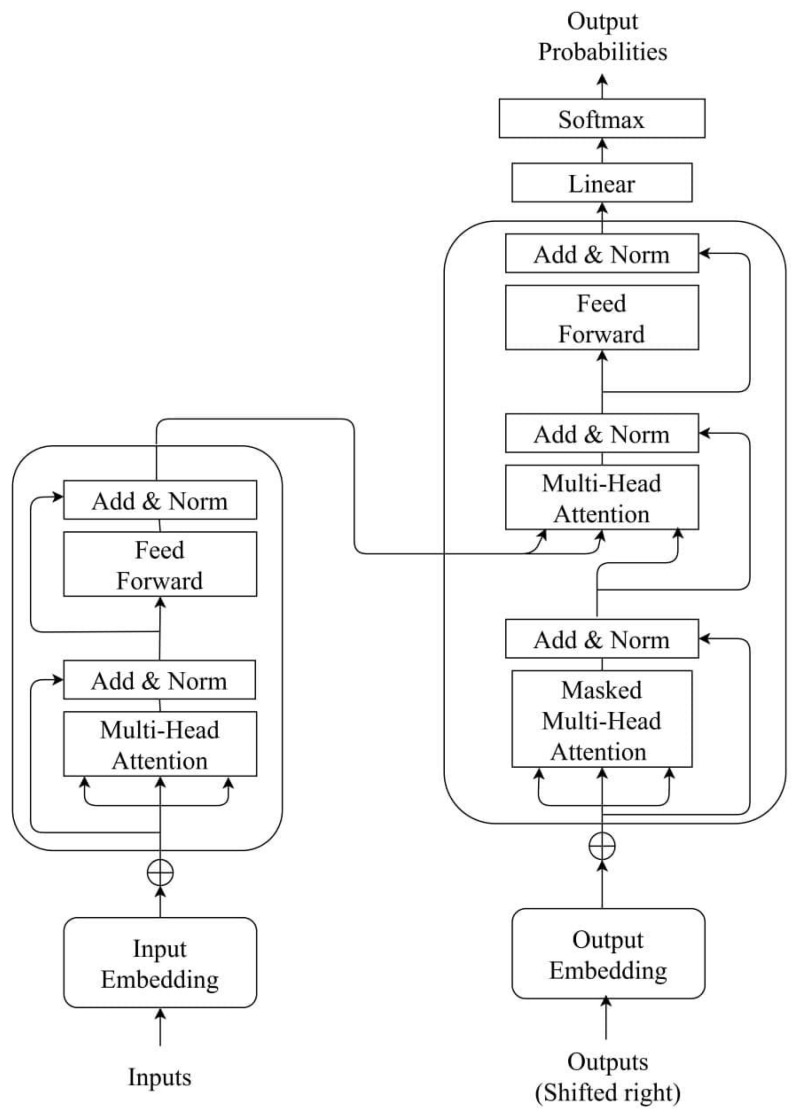
Architecture of the transformer model.

These encoder and decoder blocks are repeated n times in transformer models. One input layer and multi-head attention, dropout, and a few convolutional, normalization, and dropout layers make up the model (see Figure 3) [38]. Another input layer was included in the model to accommodate extra clinical data input (see Appendix A—Figure A10).

### 2.6. Experimental Setup

We used Python programming language with the ML frameworks Tensorflow and Keras to operate in the Google Colab platform. We tried operating with 50 and 100 epochs and batch sizes of 8, 16, and 32 and found that a batch size of 16 and 100 epochs produced the best results. The learning rate was dynamic, and it was dependent on the validation loss. The learning rate was 0.01 at first. For updating the learning rate, the patience was 5. We used early stopping with a patience of 10.

### 2.7. Statistical Analysis

Patients’ characteristics were summarized using descriptive statistics. The clinical outcomes of the patients were determined on the last days of their hospital stays as the following binary results: death (0) or discharged in stable condition (1).

The validated cutoff values for the KPS and PPI were 50% and 6.0, respectively [29]. Sensitivity, specificity, positive predictive value (PPV), negative predictive value (NPV), overall accuracy, and the area under the receiver operating characteristic (AUROC) curve were used to evaluate the predictive accuracy of the KPS and PPI. Statistical analyses were computed using Python version 3.6.

## 3. Results

### 3.1. Demographics of the Study Population

**Figure 4:**
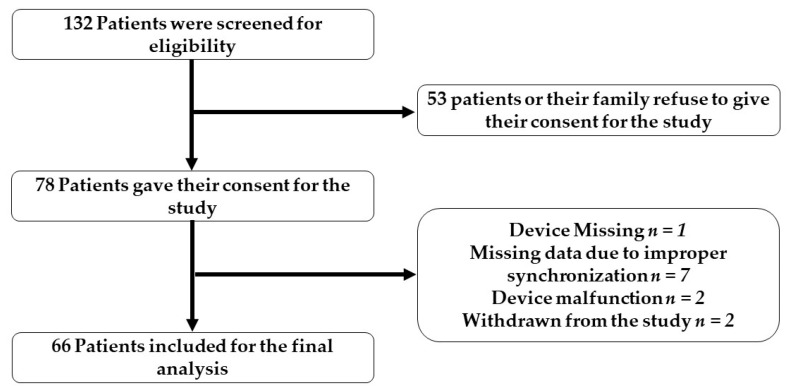
Flowchart of patient enrollment process.

Between 11 December 2019 and 30 June 2022, a total of 78 patients were enrolled during the clinical trial in the hospice care unit at TMUH. A total of 66 patients successfully completed this study, while the remaining 12 were excluded due to incomplete data or failure to synchronize the devices with their smartphones, resulting in missing data (see Figure 4). Of those 66 patients, 39 were male and 27 were female. The patients’ ages ranged from 39 to 92 years old, with a mean age of 71.42 years. The study population was predominantly male, representing 59.09% of the patients. The 2 most common types of cancer among the patients recruited for this study were colorectal cancer and non-small-cell lung cancer, accounting for 22.72% and 19.69% of all cases, respectively. The primary reason for admission to the hospice care unit was cancer-related symptoms, which affected 60.60% of the patients. Concomitant diseases were also a common reason for admission, with no patients being admitted for non-medical reasons. Concomitant diseases or symptoms were defined as those that were less related to cancer, such as infections of the lungs or urinary tract, general weakness, and changes in consciousness. The average hospital stay for patients was 11.59 days, with 35 patients being discharged in stable condition and the remaining 31 patients passing away in the hospice ward. Sedative medications were widely used to manage symptoms, such as insomnia, delirium, and restlessness, in terminally ill patients. Of the 78 patients, 36 required sedatives for less than 30% of their hospital stay, while the other 30 required sedatives for more than 30% or even greater than 70% of their stay.

Patients in palliative care often receive opioids to manage pain and relieve dyspnea. The recommended strategy for pain control involves limiting breakthrough pain to three times a day and administering a single dose of opioids that is about one-sixth of the daily dose [42,43]. During their hospital stays, patients were considered to have increased their opioid use if it was over 50% of their previous daily dose. Of the 66 patients in this study, 40 required an increase in opioids, while 24 remained stable, and 2 required a decrease. Antipyretics are used to alleviate fever caused by infection or cancer, and 51 patients had limited or no use of antipyretics (less than 30% of the time). Upon admission, each patient’s Karnofsky Performance Scale (KPS) and Prognosis Performance Scale (PPS) scores were evaluated by the physicians. The KPS and PPS scores ranged from 70 to 10, and none of the patients scored higher than 80 (see Table 1).

### 3.2. Prognostic Accuracy of KPS and PPI

Table 1 shows the absolute numbers of true positives, false positives, false negatives, and true negatives of the KPS and PPI evaluations. Overall, the KPS showed better accuracy than the PPI. We adopted a validated cutoff value of 50% for the KPS and a cutoff value of 6.0 for the PPI. True positives (discharge in stable condition) were defined as patients with KPS scores of ≥50% or PPIs of ≤6.0 at baseline visits and death at the end of their hospital stays. Based on the outcomes, the KPS had an overall predictive accuracy of 0.8333 (0.74–0.923), with a sensitivity and specificity of 0.853 (0.710–0.945) and 0.813, respectively (0.656–0.921). The NPV and PPV for the KPS were 0.829 (0.683–0.928) and 0.839 (0.685–0.939). The AUC for the KPS was 0.9 (with a 95% confidence interval of 0.826–0.974). The predictive performance of the PPI based on the binary outcomes showed an overall predictive accuracy of 0.6515 (0.5365–0.7664), with a sensitivity and specificity of 0.688 (0.517–0.829) and 0.875 (0.707–0.967), respectively. The NPV and PPV for the KPS were 0.880 (0.718–0.969) and 0.677 (0.503–0.823). The AUC for the KPS was 0.87 (with a 95% confidence interval of 0.826–0.974).

### 3.3. Training of Survival Prediction Models

**Figure 5:**
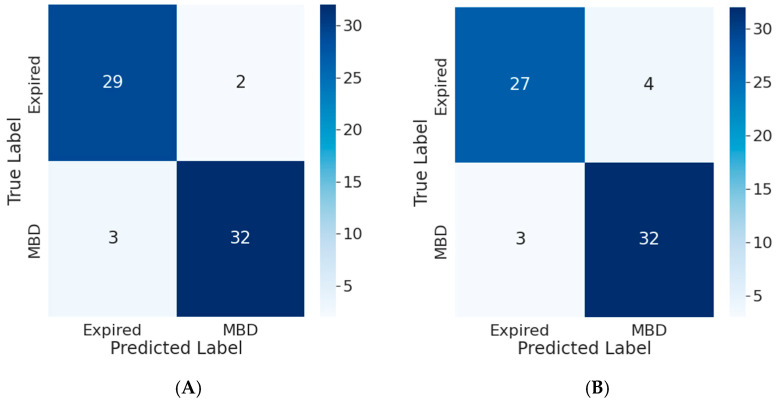
Confusion matrix for transformer model using (**A**) wearable data and (**B**) clinical and wearable data.

**Figure A10:**
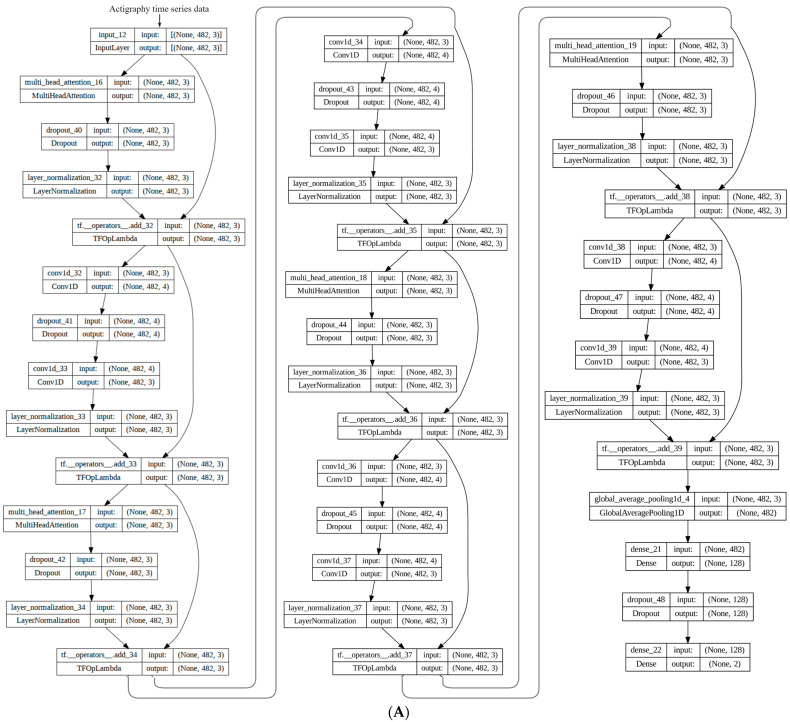

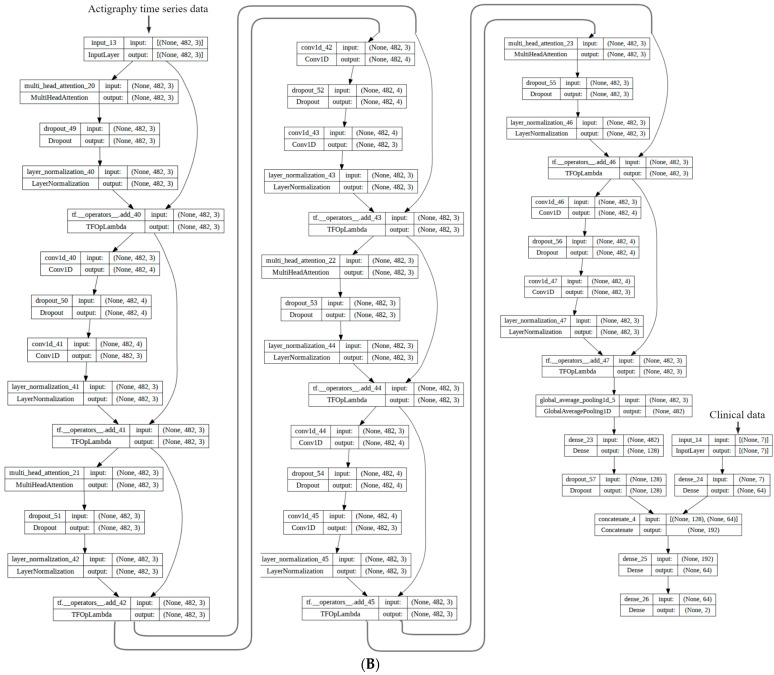
Configuration of transformer model. (**A**) Time series data and (**B**) time series and clinical data.

**Figure A11:**
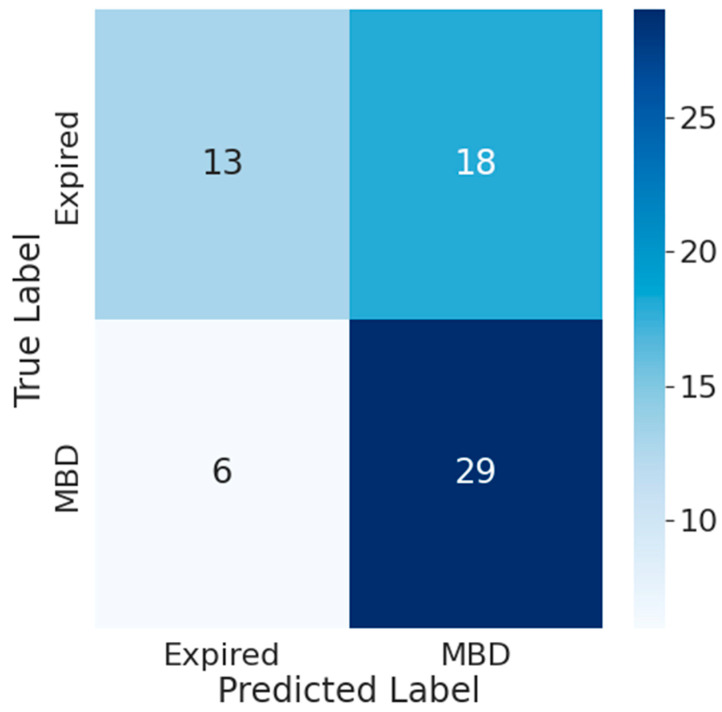
BiLSTM model confusion matrix using the wearable data.

**Figure A12:**
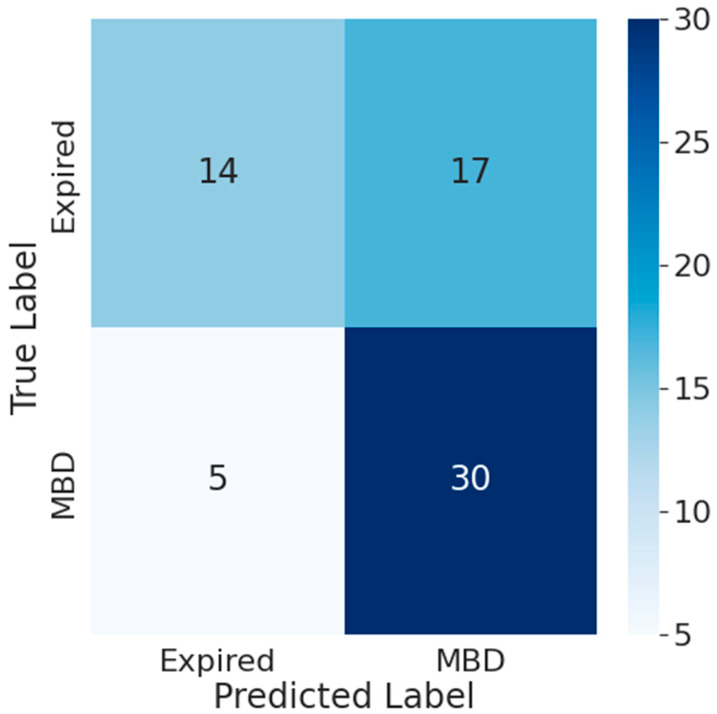
BiLSTM model confusion matrix using the clinical and wearable data.

**Figure A13:**
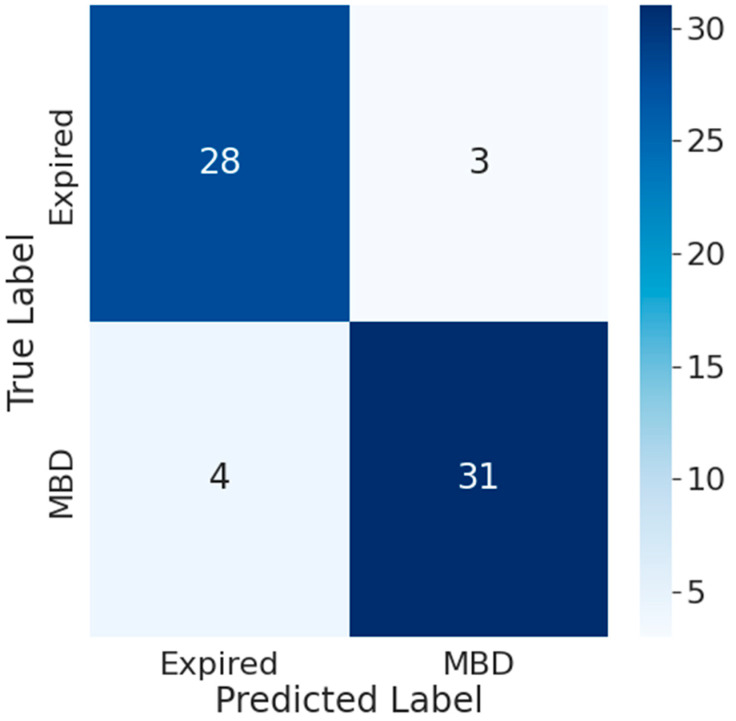
GRU model confusion matrix using the wearable data.

**Figure A14:**
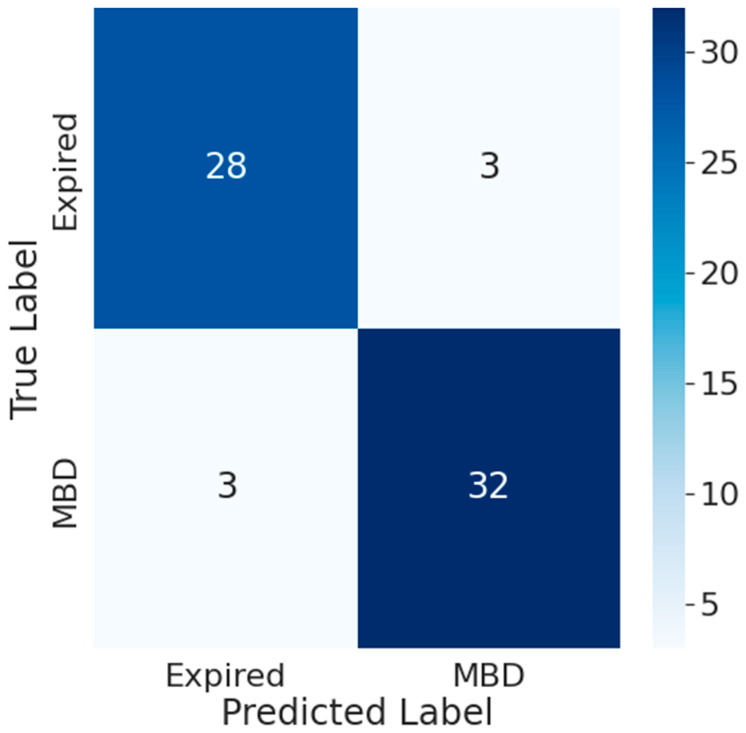
GRU model confusion matrix using the clinical and wearable data.

**Figure A15:**
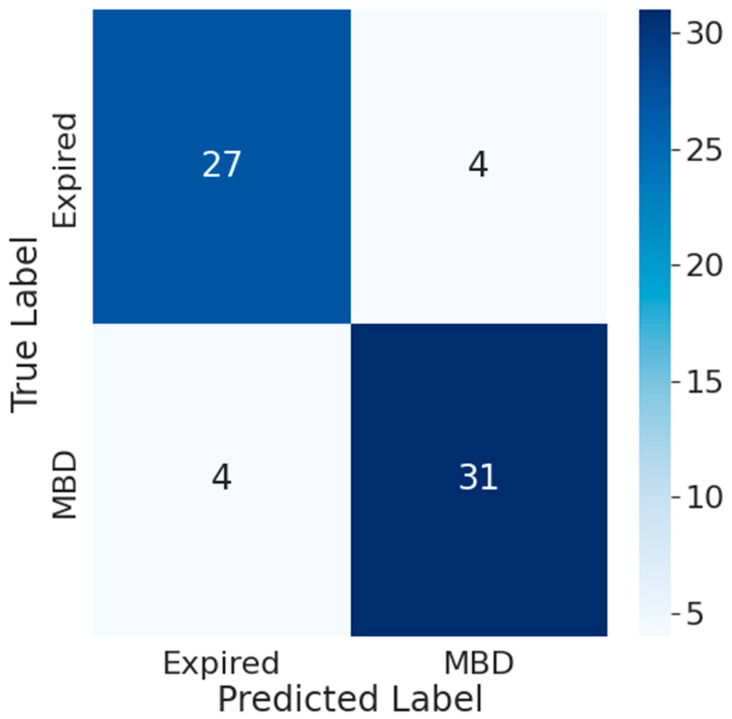
LSTM model confusion matrix using the wearable data.

**Figure A16:**
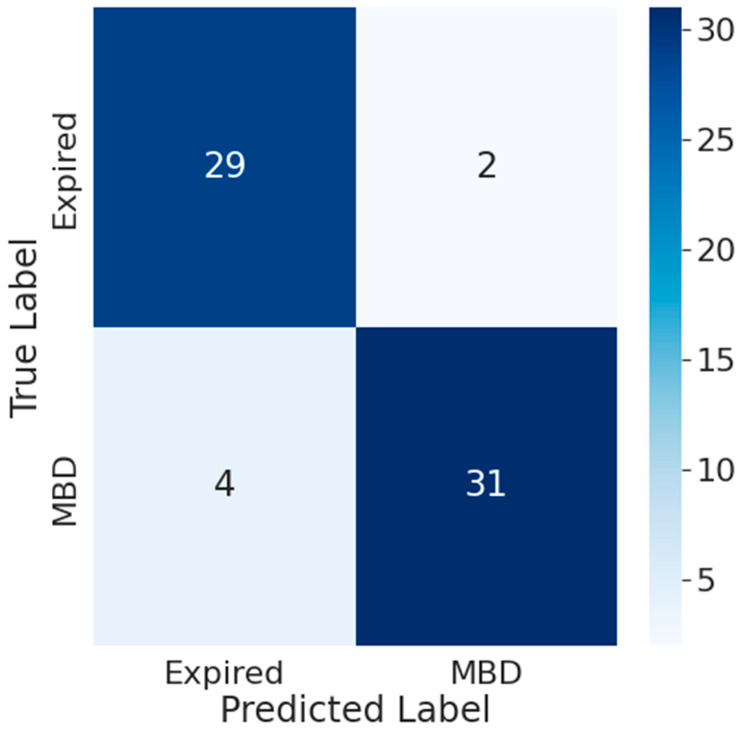
LSTM model confusion matrix using the clinical and wearable data.

Each ML model produced an accuracy of more than 0.60 based on 48 h of wearable activity data and clinical data collected after admission. The transformer model produced the best prediction for survival outcomes based on wearable and clinical data collected in 48 h. The confusion matrix for the transformer model represented the disparities between model prediction and ground reality. The variables were the same for the original and normalized confusion matrices. The sum of each row indicated the right prediction in terms of probability (see Figure 5A,B). The confusion matrices for the other models are presented in Appendix A (Figure A10, Figure A11, Figure A12, Figure A13, Figure A14, Figure A15 and Figure A16).

The transformer model that was trained using time series and clinical data had the highest accuracy, which was 0.924 (see Table 2). The transformer and GRU models provided the data with the maximum sensitivity, which was 0.914. The transformer and LSTM models yielded the highest specificity, which was 0.935. The transformer model provided us with the highest PPV and NPV scores, which were 0.941 and 0.906, respectively. We obtained the highest AUC score (0.947) using the LSTM model (see Table 2). It was clear from this study that the models trained with both time series and clinical data performed far better than those trained simply with time series data. The transformer model surpassed all the other models in terms of overall performance.

### 3.4. Impact of Time Frame on Transformer Model Performance

Since the activity data and clinical data of the initial 48 h gave the best results, we further explored the transformer model performance based on the different time intervals of 12 h and 24 h data (see Table 3). We demonstrated the performance of the transformer model for 12 and 24 h data in Table 3. The test accuracy was maintained across 24 h both for the wearable-only data and wearable plus clinical data. However, the test accuracy decreased for the 12 h data (see Table 3).

### 3.5. Comparison between the Accuracy of Traditional Prognostic Tools and Wearable Data

Wearable data showed better accuracy when compared with traditional prognostic tools. The accuracy of the wearable data was 0.893, whereas the combined accuracy of the wearable and clinical data was 0.924. The traditional tools showed accuracies of 0.8333 (KPS) and 0.6515 (PPI) (see Table 4).

## 4. Discussion

Our study utilized advanced DL techniques such as LSTM, a transformer, BiLSTM, and GRUs to predict patient survival outcomes and evaluated their performances. The results reveal that incorporating both clinical and wearable data led to improved prediction accuracy, and the DL-based models outperformed those based on prognostic tools. These findings indicate that wearable technology combined with clinical information could enhance the prognosis of end-stage cancer patients receiving hospice care. In our study, it was found that while KPS showed a similar performance, PPI produced inaccurate prognostic results and relied on highly skilled medical professionals; conversely, the proposed activity monitoring and survival prediction model did not require any clinical expertise. Since all models were tested on 48 h of data, the transformer model performed the best, with an accuracy of 0.924. Thus, we further investigated the effect of the time frame on the accuracy by analyzing patient survival prediction using activity data collected over 12 and 24 h.

The results of our current study are in line with our previous related study results, including two prospective observational studies [28,29] and a scoping review [44]. One of our previous findings showed that the majority of the included studies in the scoping review, which utilized wrist-worn wearable devices in cancer populations, focused on physical activity, sleep analysis, and heart vital signs and showed a positive correlation between patient-reported and wearable outcomes [44], while in the other study, automatic survival prediction using an LSTM DL model showed feasibility in clinical settings and possible benefits in end-of-life care settings without healthcare professionals [29]. Additionally, in the third study, wearable devices reported greater angle and spin movements as early as within the first 48 h of observation in the cancer patients who were still alive after discharge from the hospice inpatient unit [28]. Based on our previous research findings that showed the potential of wearable devices and the utility of actigraphy data as a prognostic tool for patients in hospice care, the current study builds on the concept of utilizing wearable data to predict survival outcomes in hospice patients [28]. In contrast with our previous similar work [29], wherein we utilized LSTM DL techniques for predictive analysis, the current study employed a transformer model, which yielded more noteworthy results than LSTM. Our current study provides a more comprehensive and conclusive analysis of the trial. Specifically, we included a larger patient population (see Table 1) with more detailed characteristics. We also added additional clinical features for the model building such as medication usage (including opioids, antipyretics, and sedatives) and admission causes (whether cancer-related or not), which have significant clinical implications for the prediction of survival outcomes.

While previous studies [45,46,47] have employed DL models to predict survival outcomes using electronic health records and imaging data, the current study focused on the use of wearable data as a key feature for such predictions, thus representing a novel approach in the field. Interestingly, we found that DL models are not commonly utilized for prediction analysis in the literature, as we came across only a few studies that applied these models for this purpose. For example, a study by She et al. [45] used DL models to predict survival outcomes based on histopathology images. In contrast with this, our study used continuous monitoring data for survival analysis, which is, comparatively, more effective in terms of better prognosis. A limited number of studies, such as those by Dai Xin et al. [46] and Yang Linlin et al. [47], have employed DL models to predict survival outcomes among end-stage cancer patients using electronic health records. In comparison with these studies, our research yielded more promising results by utilizing continuous monitoring data along with clinical information to predict survival outcomes among hospice care patients. Similar to our findings, another study measured rest and sleep parameters using actigraphy devices for advanced cancer patients, and after utilizing them in ML models, it was found that these sleep–wake parameters could be useful for prognostication in those patients when they were combined with routinely collected data [4]. Likewise, a few more studies have reached similar conclusions on survival prediction using ML techniques, e.g., one study showed that an ANN model provided better outcomes than logistic regression for survival prediction in cancer patients and highlighted the model’s worth as an important statistical method [24]. Another study showed how ML algorithms accurately identified cancer patients with a risk of 6-month mortality in comparison with the traditional logistic regression model and proved the importance of ML models in facilitating timely conversations between patients and healthcare providers for the required specific goals [23]. Our study aligns with these findings, indicating that machine learning models may offer superior prognostic capabilities in oncology compared with traditional statistical methods, which are not as precise at predicting cancer prognosis. In the latest systematic review of ML in palliative care [48], Vu et al. concluded that although ML in palliative care is often used to predict mortality, it is not restricted only to this purpose, as the recent literature in this domain shows the potentials of ML for other innovative use cases, e.g., for data annotation and predicting complications, as well. The authors also emphasized the need for more rigorous testing of the models to ensure their applicability in different clinical settings.

There were a few challenges and limitations associated with our study. One of the major challenges we encountered was discontinuity in the data due to battery issues in the devices or synchronization problems during showering time, as the devices were not waterproof. However, we were able to resolve this issue during the pre-processing stage [29]. In order to maintain consistency, a fixed length was applied to all patients’ time series data, which involved the shortening or zero padding of the data. While this approach ensured consistency, it could also have resulted in loss of information or distortion of the original data. Another limitation of our study was that it focused on predicting binary survival outcomes rather than the short-term or estimated survival times of patients (e.g., 15 days or 30 days). Accurately predicting survival times is important because it can help clinicians prioritize their resources and provide appropriate care for each patient. For instance, patients with a low predicted survival time may benefit more from palliative care, while those with a higher predicted survival time may benefit more from aggressive treatment. Therefore, predicting survival times along with binary outcomes could provide valuable insights for making informed clinical decisions about treatment and end-of-life care. Finally, the DL approach utilized in our study is considered a ‘black box’ due to the lack of understanding of its mechanisms of operation. This difficulty in interpretation is a common challenge associated with deep learning models, as it makes it challenging to comprehend the underlying reasons for predictions. Despite the lack of clarity, this approach demonstrated promising results in the context of our study.

## 5. Conclusions

This study addressed the critical need for accurate survival estimates in hospice care for terminal cancer patients by developing a deep learning (DL)-based model that predicts survival outcomes using actigraphy data and clinical information. The results of this study show that the transformer model produced an accuracy of 0.924 based on 48 h of wearable activity data and clinical data collected after admission. The models trained with both time series and clinical data produced better results than those trained only with time series data. In particular, the transformer model showed the best prediction accuracy of 0.878 and 0.924 for survival outcomes based on sensor and clinical data collected over a 12 and 24 h period. These findings suggest that a short data collection period of 24 or 48 h is sufficient for making accurate predictions.

The implications of this study are significant for the future of end-of-life care. The use of wearable technology and deep learning models for predicting survival outcomes can provide healthcare providers with more objective and accurate information for decision making. This, in turn, can lead to better support for patients and their families in the end-of-life care process. In addition, the integration of wearable technology and deep learning models can contribute to the development of personalized and patient-centered end-of-life care plans. By understanding a patient’s individual needs and predicting their likelihood of survival, healthcare providers can create tailored care plans that meet the patient’s specific goals and wishes. Furthermore, the use of wearable technology and deep learning models can also improve the efficiency of healthcare delivery in end-of-life care. With continuous patient monitoring, healthcare providers can detect changes in a patient’s condition and intervene earlier, leading to better outcomes and reduced hospitalizations.

## Figures and Tables

**Table 1:** Characteristics of recruited patients.

**Total recruited patients, *n* =**		78
**Patients analyzed, *n =***		66
**Age (years); mean (range)**		71.42 (39–92)
**Gender, *n* = (%)**		
Male		39 (59.09%)
Female		27 (40.90%)
**Primary site of cancer, *n* =**		
Bladder cancer		1
Brain cancer		2
Breast cancer		3
Cervical cancer		2
Cholangiocarcinoma		1
Colorectal cancer		15
Duodenal cancer		1
Endometrial cancer		1
Esophageal cancer		3
Gastric cancer		5
Hepatocellular carcinoma		2
Head and neck cancer		3
Lung cancer (non-small-cell)		13
Small-cell lung cancer		1
Ovarian cancer		2
Pancreatic cancer		2
Prostate cancer		8
Vaginal cancer		1
**Admission cause; *n* = (%)**		
Concomitant diseases		26 (39.39%)
Cancer-related symptoms		40 (60.60%)
**Duration (days); mean (range)**		11.59 (0–41)
**Outcome, *n* = (%)**		
Discharge		35 (53.03%)
Death		31 (46.96%)
**KPS score, *n* =**		
Positive (KPS ≥ 50%)	TP (29)	FN (5)
Negative (KPS < 50%)	FP (6)	TN (26)
**PPI score, *n* =**		
Positive (PPI ≤ 6)	TP (22)	FN (10)
Negative (PPI > 6)	TP (3)	FN (21)
**Use of sedatives (time of study days), *n* =**		
<30%		36
30–70%		8
>70%		22
**Status of using opioids, *n* =**		
Decreasing use		2
Stable		24
Increasing use		40
**Use of antipyretics (time of study days), *n* =**		
<30%		51
30–70%		12
>70%		3

KPS: Karnofsky Performance Scale; PPI: Palliative Prognostic Index.

**Table 2:** Performances of different models using the wearable data and the combined data of wearable and clinical data from 48 h data.

Model	Dataset	Accuracy	Sensitivity	Specificity	PPV	NPV	AUC
LSTM	Wearable only	0.878	0.885	0.87	0.885	0.870	0.920
	Wearable + clinical	0.909	0.885	0.935	0.939	0.878	0.947
Transformer	Wearable only	0.893	0.914	0.87	0.888	0.9	0.919
	Wearable + clinical	0.924	0.914	0.935	0.941	0.906	0.927
BiLSTM	Wearable only	0.636	0.828	0.419	0.617	0.684	0.795
	Wearable + clinical	0.666	0.857	0.451	0.638	0.736	0.791
GRU	Wearable only	0.893	0.885	0.903	0.911	0.875	0.877
	Wearable + clinical	0.909	0.914	0.903	0.914	0.903	0.940

LSTM: Long Short-Term Memory network; BiLSTM: Bidirectional Long Short-Term Memory network; GRU: Gated Recurrent Unit.

**Table 3:** Performance of transformer model using the wearable data and the combined data of wearable and clinical data from 12 and 24 h data.

Transformer Model		Accuracy	Sensitivity	Specificity	PPV	NPV	AUC
12 h	Wearable only	0.848	0.881	0.806	0.837	0.862	0.884
	Clinical + wearable	0.878	0.914	0.838	0.864	0.896	0.934
24 h	Wearable only	0.893	0.857	0.935	0.937	0.852	0.929
	Clinical + wearable	0.924	0.971	0.870	0.894	0.964	0.956

PPV: positive predictive value; NPV: negative predictive value; AUC: area under the ROC curve.

**Table 4:** Comparison between the accuracy of wearable and traditional prognostic tools.

Prognostic Tool		Accuracy	Sensitivity	Specificity	PPV	NPV	AUC
KPS		0.8333	0.853	0.813	0.829	0.839	0.9
PPI		0.6515	0.688	0.875	0.880	0.677	0.87
Transformer model	Wearable only	0.893	0.914	0.87	0.888	0.9	0.919
	Wearable + clinical	0.924	0.914	0.935	0.941	0.906	0.927

KPS: Karnofsky Performance Scale; PPI: Palliative Prognostic Index.

## Data Availability

The raw data supporting the conclusions of this article will be made available by the authors, without undue reservation.

## Appendix A

### Appendix A.1. LSTM

An LSTM unit contains a cell state that can selectively learn, unlearn, or retain knowledge from each of the units (see Figure A1) [39].

An LSTM unit has three gates: the input, output, and forget gates. The input (*x*) and previous cell state (*h*) go through the sigmoid function, which sets the value of information between zero and one. The less valued information is filtered through the forget gate and gives the filtered output (*i*). The hyperbolic tanh function determines the output of cell C. The model comprises an LSTM layer with a neuron unit of 256 and an L2 regularizer with an initial value of 0.01, a dense layer of 64 neurons, an L2 regularizer with an initial value of 0.01, and a ‘relu’ activation function covered with a time-distributed layer, a flatten layer, and a dense layer with 2 neurons and ‘Softmax’ activation, sequentially (see Figure A2).

There was an additional input layer with seven features for utilizing the seven types of clinical data, which passed through a dense layer and was concatenated with the previous time series input. After that, the model was built out of two successive dense layers. The window size was 482 for each time with 3 input features for the 3 types of time series data that we obtained with the actigraphy device (see Figure A3).

### Appendix A.2. BiLSTM

A bidirectional LSTM, often known as a BiLSTM, is a sequence processing model that consists of two LSTMs, one of which receives input forward and the other of which receives it backward. The model consisted of 2 layers of a bidirectional LSTM, with 128 and 64 neurons, respectively. Additionally, there were 2 dropout layers implemented to drop 10% of the neurons, followed by 2 dense layers with 64 and 2 neurons, in sequential order (see Figure A4 and Figure A5) [49].

The clinical data were used in a second input layer with seven features, which was concatenated with the first time series input after passing through a dense layer. The model was then constructed using two successive dense layers. For the time series data, the window size was always 482, with 3 input characteristics from the actigraphy device (see Figure A6).

### Appendix A.3. GRU

The update gate, reset gate, current memory unit, and final memory unit are the four main parts of a GRU (gated neural network). The weights are updated with the update gate, which also solves the vanishing gradient issue. The model continues to update the information so that it can be transmitted to the future as it learns on its own. In contrast, the reset gate decides how much of the prior knowledge should be erased in considering the current situation.

The reset gate (*r*) erases unnecessary information by using data from the previous cell state (*h*) and input (*x*). The information is updated with the update (*u*) gate. A sigmoid function is used to update and filter the data, and the output is determined via the hyperbolic tanh function. The model consisted of a GRU layer with 256 neurons, which included an L2 regularizer with an initial value of 0.01. This was followed by a dense layer wrapped with 64 neurons and ‘relu’ activation, along with a time-distributed layer, a flatten layer, and a final dense layer with 2 neurons and ‘Softmax’ activation (see Figure A7 and Figure A8) [50].

For the categorization using both sensor and clinical data, the entire model remained consistent. The initial layer of the model, which was solely utilized for time series classification, was concatenated with an input layer to receive clinical data input. An additional dense layer was employed for this purpose (see Figure A9).

### Appendix A.4. Transformer Model

We used a multi-head attention layer in the transformer model, which consists of different layers running in parallel. Encoder and decoder blocks are composed of Multi-Head Attention and Feed-Forward Networks, with the best features chosen via Feed-Forward Networks. These encoder and decoder blocks are repeated n times in transformer models. The input layer had a dimension of three-for-three time series features. The encoder part was composed of a multi-head attention layer with a key dimension of 256, 4 heads, and 25% dropout. The feed-forward part included 1 convolutional layer with 4 filters, a kernel size of 1, and ‘relu’ activation, along with 25% dropout, another convolutional layer with 3 filters, a kernel size of 1, and a normalization layer. The encoder part consisted of four blocks. The 128 multilayer perceptron units were composed of ‘relu’ activation and 40% dropout (see Figure A10A). Additionally, another input layer with 7 features was included in the model to accommodate extra clinical data input, with 2 sequential dense layers of 64 and 2 neurons with ‘relu’ and ‘Softmax’ activation, respectively (see Figure A10B).

**Results**

The confusion matrices for the LSTM, BiLSTM, and GRU models represented the disparities between model prediction and ground reality. The variables were the same for the original and normalized confusion matrices. The sum of each row indicates the right prediction in terms of probability.

See the following Figures.
